# Bax Translocation Mediated Mitochondrial Apoptosis and Caspase Dependent Photosensitizing Effect of *Ficus religiosa* on Cancer Cells

**DOI:** 10.1371/journal.pone.0040055

**Published:** 2012-07-06

**Authors:** Jazir Haneef, Parvathy M, Santhosh Kumar Thankayyan R, Hima Sithul, Sreeja Sreeharshan

**Affiliations:** Cancer Research Program, Rajiv Gandhi Centre for Biotechnology, Thiruvananthapuram, Kerala, India; Texas A&M University, United States of America

## Abstract

The main aim of the present work was to investigate the potential effect of acetone extract of *Ficus religosa* leaf (FAE) in multiple apoptosis signalling in human breast cancer cells. FAE treatment significantly induced dose and time dependent, irreversible inhibition of breast cancer cell growth with moderate toxicity to normal breast epithelial cells. This observation was validated using Sulforhodamine B assay. Cell cycle analysis by Flow cytometry showed cell cycle arrest in G1 phase and induction of sub-G0 peak. FAE induced chromatin condensation and displayed an increase in apoptotic population in Annexin V-FITC/PI (Fluorescein isothiocyanate/Propidium iodide) double staining. FAE stimulated the loss of mitochondrial membrane potential in multiple breast cancer cell lines when compared to normal diploid cells. To understand the role of Bax in FAE induced apoptosis, we employed a sensitive cell based platform of MCF-7 cells expressing Bax-EGFP. Bax translocation to mitochondria was accompanied by the disruption of mitochondrial membrane potential and marked elevation in LEHDase activity (Caspase 9). Consistent with this data, FAE induced Caspase activation as evidenced by ratio change in FRET Caspase sensor expressing MCF-7 cell line and cleavage of prominent Caspases and PARP. Interestingly, FAE accelerated cell death in a mitochondrial dependent manner in continuous live cell imaging mode indicating its possible photosensitizing effect. Intracellular generation of reactive oxygen species (ROS) by FAE played a critical role in mediating apoptotic cell death and photosensitizing activity. FAE induced dose and time dependent inhibition of cancer cell growth which was associated with Bax translocation and mitochondria mediated apoptosis with the activation of Caspase 9 dependent Caspase cascade. FAE also possessed strong photosensitizing effect on cancer cell line that was mediated through rapid mitochondrial transmembrane potential loss and partial Caspase activation involving generation of intracellular ROS.

## Introduction

Herbal plants and plant-derived medicines have been widely used in traditional cultures all over the world and have gained popularity in modern society as natural alternatives to produce new potential therapeutic compounds for combating diseases [1]. The health promoting effects of plant constituents and extracts are being increasingly studied and their consumption is on the rise [2]. Many herbs have been evaluated in clinical studies and are currently being investigated phytochemically to understand their tumoricidal actions against various cancers. Unfortunately, majority of the studies were carried out on individual molecules that were found to be less effective as chemopreventive agents compared to phytochemical cocktails that may induce their activity by synergism [3].

Pharmacological studies carried out on the fresh plant materials of *F.religiosa* provide a pragmatic support for its numerous traditional uses. Its bark, fruits, leaves, adventitious roots, latex and seeds are medicinally used in different forms, sometimes in combination with other herbs [4]. Phytochemical research carried out on *F.religiosa* had led to the isolation of phytosterols, amino acids, furanocoumarins, flavonoids, phenolic components, hydrocarbons, aliphatic alcohols, volatile components and few other classes of secondary metabolites, tannins, steroids, alkaloids and β-sitosteryl-d-glucoside, Vitamin K, n-octacosanol, methyl oleanolate, lanosterol, stigmasterol, lupen-3-one [5], [6]. Singh *et al* [7] had suggested a detailed investigation for its potential against cancer, cardiovascular, neuro inflammatory, neuropsychiatric, oxidative stress related disorders and parasitic infections. Most of the pharmacological studies were aimed on validating its traditional uses for wound healing [8], [9], anti-bacterial [10], anti-convulsant [11], anti-diabetic [12], anti-oxidant [13], anti-inflammatory [14], acetyl cholinesterase inhibitory activity [15] and anti-anxiety activity [16]. The methanolic extract of *F.religiosa* leaf exerts strong neuroprotective effect against inflammation caused by mediators such as nitric oxide and cytokines in LPS (Lipopolysaccharide)-stimulated microglia via the MAPK (Mitogen Activated Protein Kinase) pathway [17].

*Ficus species* were shown to have anti proliferative activity in tumor cell lines and its various parts have shown apoptotic effects, thereby providing a preliminary pharmacological support for their use as anticancer drug [18]. Till date, no literature and experimental evidence are available for substantiating the anti-cancer and apoptotic effect of *F.religiosa* leaf extracts on multiple breast cancer cells. This prompted us to investigate the possible mechanism of its apoptosis promoting activity and to identify additional biological activity, if any.

Cell cycle is a process that acts as a key to control growth and proliferation of a cell. The disruption of the cell cycle process will cause an imbalance between cell proliferation and cell death, subsequently leading to cancer development. Thus, cell cycle could serve as target for anticancer agents to halt uncontrolled proliferation of tumor cells and to initiate them to undergo apoptosis [19]. The apoptotic process (or programmed cell death) is an important mechanism in response to cytotoxic treatment and its induction is a highly desirable *modus operandi* for an anticancer agent [20]. One of the challenges in cancer treatment is that malignant cells possess the ability to evade apoptosis, which is the major cause for the ineffectiveness of any cytotoxic drug to kill such cells. The present study shows the effect of acetone extract of *F.religiosa* leaves on the dose and time dependent growth inhibition of multiple breast cancer cell lines which was associated with Bax translocation and mitochondria mediated apoptosis with the activation of Caspase 9 dependent pathway. Even though FAE induced significant Caspase activation both in enzymatic assay as well as in live cell Caspase sensor cell models, the continuous exposure of the treated cells revealed an unexpected photosensitizing activity**.** This study is important because it is the first report providing evidence to show that bio-available constituents of *F.religiosa* leaf extract exert photosensitizing and apoptosis inducing capability through the generation of intracellular ROS.

## Results

### Phytochemical Analysis

The freshly prepared crude acetone extract of *Ficus* leaves (FAE) was qualitatively tested for the presence of alkaloids, flavonoids, phenols, saponins and tannins (Table 1) using standard procedures of analysis [21]. Aluminium chloride colorimetric method was used for flavonoids estimation [22]. The flavonoid content of the extract in terms of quercetin equivalent was 79±4 mg/g of dry FAE powder. The total phenols estimated by Folin Ciocalteu method [23] in terms of gallic acid equivalent was 110±2.18 mg/g in the extract powder.

**Table 1:** Preliminary phytochemical analysis of Leaf extract of *Ficus religiosa* revealed the presence of alkaloids, flavanoids, phenols, sterols and tannins.

Sl.No.	Phytochemical	Occurrence
1.	Alkaloids	+
2.	Flavonoids	+
3.	Glycosides	−
4.	Saponins	−
5.	Phenols	+
6.	Sterols	+
7.	Tannins	+

‘+’ =  present, ‘−’ =  absent.

### FAE Inhibited Proliferation of Breast Cancer Cell Lines

**Figure 1:**
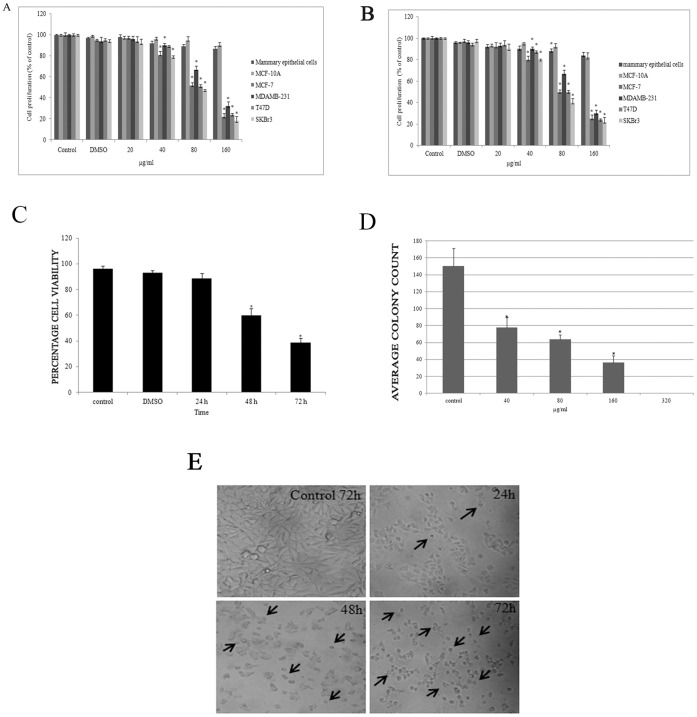
Effect of FAE on the proliferation and viability of MCF-7 and MCF-10A cells. (**A**) MCF-7 cell proliferation was assessed by MTT assay- Cells were treated with 20, 40, 80, 160 and 320 µg/ml of FAE for 48 h. IC_50_ value for each cell lines determined from the MTT data are- 363.6 µg/ml (Mammary epithelial cells), 800 µg/ml (MCF10A), 83.3 µg/ml (MCF-7), 121.2 µg/ml (MDAMB231), 81.6 µg/ml (T47D) and 75.47 µg/ml (SKBr3). (**B**) Cytotoxicity was determined using a protein based viability test Sulforhodamine B assay (**C**) Cell viability by trypan blue dye exclusion assay, MCF-7 cells were treated with IC_50_ of FAE for 24, 48 and 72 h. The data shown represents mean ± SD of three independent experiments. (**D**) FAE inhibits colony formation of breast cancer cells. MCF-7 cells were seeded in six well plates at 500 cells/well in phenol red free DMEM containing 10% FBS. After 12 h, cells were treated with different concentrations of FAE. The medium with FAE was changed after every 4 days. After 14 days of incubation, colonies were stained with 0.3% crystal violet solution for 2 mins, washed with PBS, air-dried and counted. Each experiment was performed in triplicates. (**E**) Growth inhibition and morphologic changes of MCF-7 cells treated at IC_50_ value of FAE for 24, 48 and 72 h, compared with untreated cells. Cells were photographed with a Leica DMIL inverted microscope (200×).

The anti-proliferative effect of FAE on the growth of Mammary epithelial cells, MCF-10A, MCF-7, MDAMB231, T47D and SKBr3 cells were initially determined by MTT (3-(4, 5-Dimethylthiazol-2-yl)-2, 5-diphenyltetrazolium bromide) assay and cytotoxicity by trypan blue dye exclusion assay. Cells were treated with increasing concentrations of FAE (20–320 µg/ml) for 24, 48 and 72 h. FAE inhibited the growth of breast cancer cells in a dose and time dependent manner (Figure 1A). IC_50_ value for each cell lines determined from the MTT data are - 363.6 µg/ml (Mammary epithelial cells), 800 µg/ml (MCF10A), 83.3 µg/ml (MCF-7), 121.2 µg/ml (MDAMB231), 81.6 µg/ml (T47D) and 75.47 µg/ml (SKBr3). In the two non-tumorigenic mammary epithelial cells, FAE showed moderate cytotoxicity than cancer cells. A protein based Sulforhodamine B assay was also performed that substantiated the MTT results (Figure 1B). These results suggested that FAE showed great selectivity towards cancer cells than normal cells. In addition, microscopic observation on cell morphology revealed that FAE treatment induced noticeable morphology alterations including blebbing of membrane and shrinkage of cells apart from reduction in cell density in a time dependent manner (Figure 1E) which was consistent with the trypan blue dye exclusion data (Figure 1C). We have performed clonogenic cell survival assay to assess the effect of FAE on colony formation. FAE treatment significantly decreased the number of colonies in a dose dependent manner in MCF-7 cells (Figure 1D).

### FAE Caused Mild Cell Cycle Arrest and Sub-Go Induction

**Figure 2:**
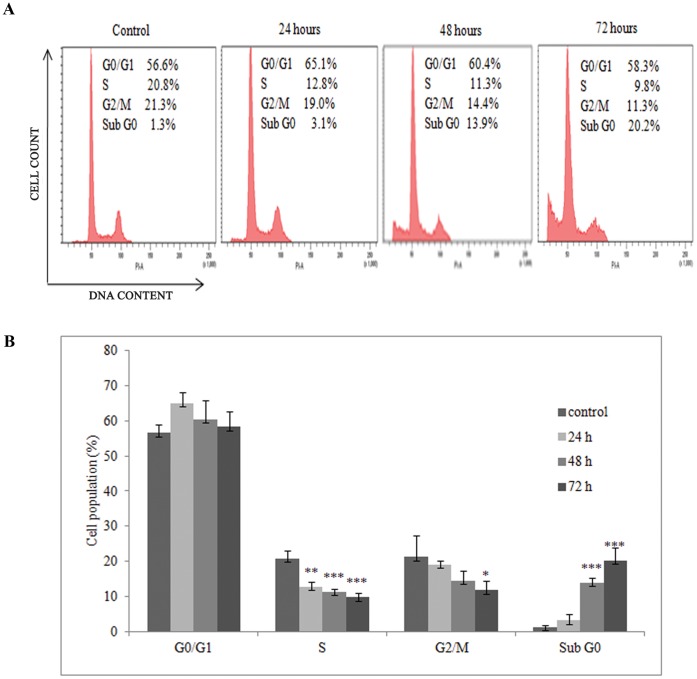
Effect of FAE on cell cycle. MCF-7 cells were cultured with FAE at 100 µg/ml value for 24, 48 and 72 h and the cell cycle phase distribution was determined by PI staining and analyzed using BD Diva software (**A**)**.** The percentage of cell cycle phases are shown in the histogram (**B**)**.**

Cell viability assays confirmed the ability of FAE to inhibit MCF-7 cell growth. Cell cycle analysis using flow cytometry was carried out to determine whether the FAE induced inhibition of MCF-7 cell growth was the result of induction of apoptosis or cell cycle arrest or the simultaneous activation of these two modes. A representative histogram along with enclosed data is given (Figures 2 A and B). The results revealed that FAE treatment at 100 µg/ml for 24, 48 and 72 h induced a dose and time dependent increase in the percentage of cells in sub- G_0_ phase, which was accompanied by a corresponding reduction in the percentage of cells in S and G_2_/M phase. On 24 h exposure, there was no considerable alteration in the cell cycle phase distribution. Treatment with FAE for 48 and 72 h increased the cell population of G_1_ phase to 60.4% and 58.3% respectively as compared to control 56.6% (Figures 2 A and B). All these findings indicated that FAE induced mild G_1_ phase arrest and induction of sub-Go population. All values were obtained from three independent experiments. Significant differences from control value was indicated by *(p<0.05), **(p<0.01) or ***(p<0.001).

### FAE Induced Chromatin Condensation and Apoptosis

**Figure 3:**
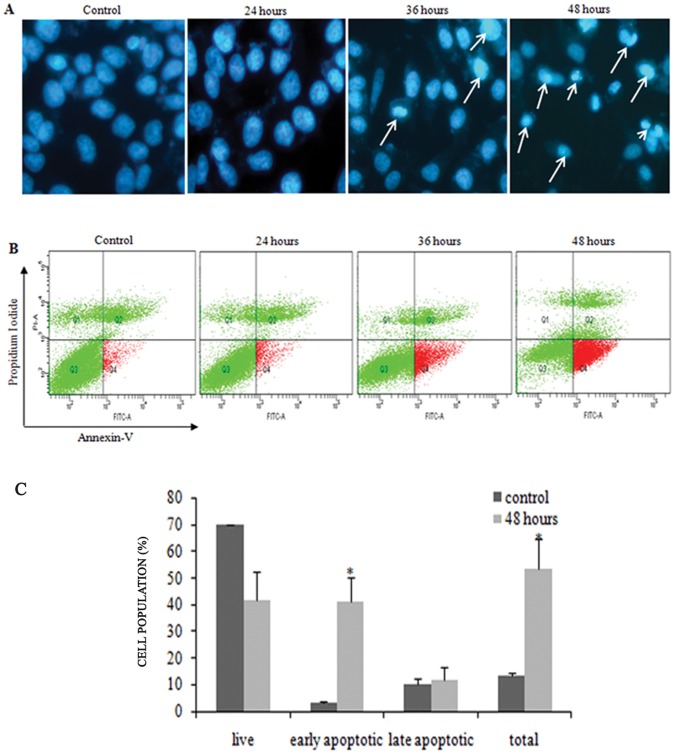
*In vitro* assessment of apoptosis in MCF-7 cells induced by FAE. (**A**) Nuclear changes associated with MCF-7 cells treated with IC_50_ value of FAE after Hoechst 33342 staining under Eclipse E-600 fluorescence microscope (400×). The number of cells showing characteristic apoptotic morphology emitting bright fluorescence increased in a time dependent manner**.** (**B**) FACS analysis via Annexin V-FITC/PI staining was used to observe the induction of apoptosis. Cells in the lower right quadrant indicate Annexin-positive/PI negative, early apoptotic cells. The cells in the upper right quadrant indicate Annexin-positive/PI positive, late apoptotic or necrotic cells. (**C**) The percentage of cells undergoing early and late apoptosis in comparison with the respective control on treatment with FAE for 48 h, at IC_50_ value. Each value represents mean ± SD of three independent experiments. Significant difference from control value was indicated by *(p<0.05).

Hoechst 33342 staining of MCF-7 cells treated with FAE at IC_50_ for 24, 36 and 48 h also showed the appearance of characteristic apoptotic changes such as condensation of nuclear chromatin (Figure 3A). FAE induced apoptosis was further confirmed by Annexin V-FITC/PI double staining. The cellular changes involved in the process of apoptosis included loss of phospholipid asymmetry. At the onset of apoptosis, phosphatidylserine, which is normally found on the inner layer of plasma membrane, becomes translocated to the exterior. The Annexin V-FITC can bind to the exposed phosphatidylserine on the surface of the plasma membrane [24]. Annexin V-positive/PI negative cells were considered early apoptotic, Annexin V positive and PI positive cells were late apoptotic or necrotic. There was no major increase in early or late apoptotic cell population on 24 h treatment with FAE (Figure 3B). However, following 36 h of treatment, the percentage of early apoptotic cells increased to 16.85% as compared to control 3.15%. After 48 h of treatment, the total apoptotic population increased up to 53.4% (41.4% early apoptotic and 12% late apoptotic, p<0.05; Figure 3C).

### FAE Stimulated Translocation of Bax-GFP to Mitochondria and Loss of Mitochondrial Transmembrane Potential in a Time Dependent Manner

**Figure 4:**
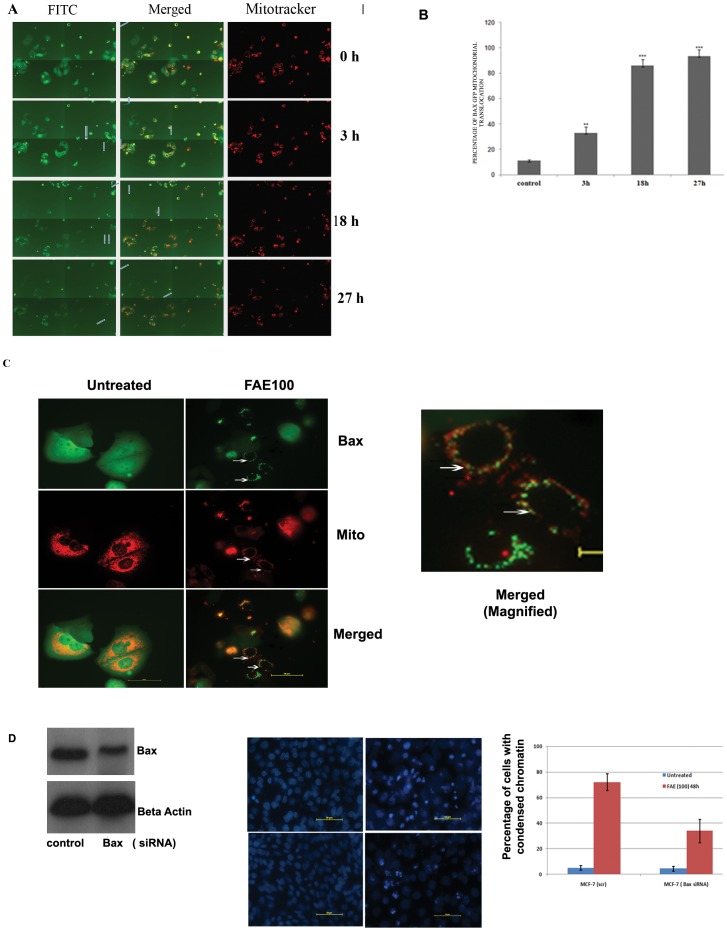
Effect of FAE in regulating Bax translocation. (**A**) MCF-7 cells expressing Bax EGFP and Mito DsRed were seeded in 96 well glass bottom plate (BD, USA) with low density and after 48 h, treated with FAE at 100 µg/ml. The image was taken using BD Pathway Bioimager 435 (Becton Dickinson, USA) at 3, 18 and 27 h by setting Montage (2×2) and specific Macro using AttoVision™ software. The green granular aggregate indicates the translocation of Bax to mitochondria, as indicated by the arrows. The representative images collected at indicated time points were used for analysing the percentage positive cells with Bax EGFP onto mitochondria compared to total in the field. (**B**) Graph showing the percentage of cells undergoing Bax translocation into mitochondria upon FAE treatment. (**C**) The MCF-Bax-DS Red cells were treated as indicated above. Bax-EGFP accumulation in mitochondria is indicated in high magnification images with arrows. A magnified merged image of the treated cells is also shown. (**D**) MCF-7 cells were transfected with Control (scr) siRNA or Bax siRNA. After 48 h of transfection, whole cell extract was prepared and immunoblotted for Bax and Actin. The same cells were also stained with Hoechst 33342 after 48 h of FAE treatment to visualize chromatin condensation (left panel). The graph is the quantitative representation of the percentage of cells with condensed chromatin in scrambled-transfected and Bax-transfected cells after FAE treatment.

**Figure 5:**
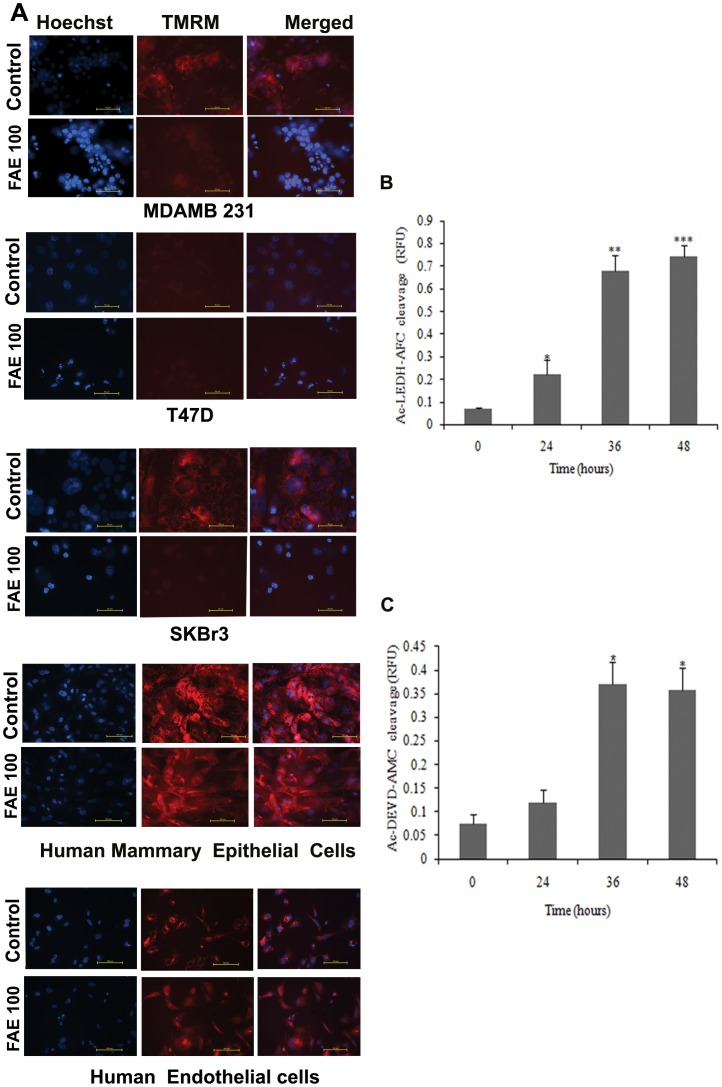
Effects of FAE on mitochondrial membrane potential and Caspase activation in breast cancer cells and normal cells. (A) The breast cancer cells, MDAMB231, T47D, SKBr3 and normal cells like Human Mammary Epithelial cells and Human Endothelial cells after treatment with FAE at 100 µg/ml for 24 h were stained with 50 nM of TMRM and 0.5 µg/ml of Hoechst 33342 for 15 mins. Then the cells were imaged under fluorescent microscope using DAPI and Rhodamine filter sets using 40× objective. The images were captured with Retiga Exi camera using NIS element software (Nikon). (B) Ac-LEHD-AFC cleavage (Caspase 9 activity) (C) Ac-DEVD-AMC cleavage (Caspase 3/7 activity). MCF-7 cells were treated at IC_50_ value of FAE for 24, 36 and 48 h. The results were measured fluorometrically. Values are expressed as mean ± SD of triplicate samples. Significant difference from control value was indicated by *(p<0.05), **(p<0.01) or ***(p<0.001). FAE treatment resulted in a time dependent increase in the cleavage of Ac-LEHD-AFC (Acetyl-Leu-Glu-His-Asp-7-amino-4-trifluoromethyl coumarin) suggesting increased activity of Caspase 9 (Figure 5B). On 24 h treatment, no prominent cleavage of Ac-DEVD-AMC (substrate for Caspases 3/7; Acetyl-Asp-Glu-Val-Asp-7-Amino-4-methylcoumarin) was observed. Noticeable DEVDase activity occurred only at 36 h treatment with FAE. Scale bar represents 50 micrometers.

Bax is a strong multi-domain pro-apoptotic protein that resides in the cytoplasm as inactive monomer in healthy cells. Upon apoptotic stimuli, Bax undergoes conformational activation leading to its translocation to mitochondria. Several studies had previously implicated role of Bax and its conformational activation as the early events that contribute to the release of Cytochrome c from mitochondria and subsequent Caspase activation in drug induced intrinsic apoptosis signalling [25]. We have employed a sensitive cell based platform, MCF-7 cells expressing Bax-EGFP (Enhanced Green Fluorescent Protein) and Mito DsRed to detect Bax translocation to mitochondria in FAE induced cell death. In order to score activity in large number of live cells, Pathway Bio imager was used with 2×2 montages as described [26]. The representative image of MCF-7 Bax EGFP cells after treatment with 100 µg/ml of FAE was shown in Figure 4A. As shown in Figure 4B, as early as 3 h of drug treatment nearly 33.3% cells showed granular mitochondrial pattern in the treated cells compared to untreated cells. Before FAE treatment, the Bax-EGFP showed diffused cytosolic pattern indicating their monomeric status. In later hours, majority of cells showed granular pattern with massive large Bax oligomers on mitochondria (Figure 4B). A high magnification image of Bax aggregates in mitochondria is also shown in Figure 4B. We have observed an early increased Bax translocation in Bax over-expressing cells that was consistent with previous report that over-expression of Bax sensitized cells to death because of its inherent pro-apoptotic activity [27]. The result clearly substantiated that early Bax activation played a critical role in FAE induced apoptosis signalling. Silencing of Bax using siRNA reduced the chromatin condensation in FAE treated cells as compared to scrambled sequence transfected cells (Figures 4 ii and iii). Further, we had utilized additional three breast cancer cell lines, MDAMB 231, SKBr3, T47D and two normal diploid cells to profile apoptotic activity of FAE. To visualize the mitochondrial membrane potential and chromatin condensation simultaneously, cells after treating with 100 µg/ml of FAE for 36 h were stained with mitochondrial membrane potential specific dye TMRM and nuclear stain Hoechst 33342 as described [28]. As shown in Figure 5A, most of the treated breast cancer cells showed decrease in TMRM intensity indicating that mitochondrial membrane potential was lost that well correlated with condensed chromatin. Interestingly both human endothelial cells as well as human mammary epithelial cells showed less TMRM intensity loss and less chromatin condensation than the cancer cells. The results suggested that FAE induced cell death in most breast cancer cells with loss of mitochondrial membrane potential and chromatin condensation. The normal proliferating diploid cells were relatively resistant to apoptotic cell death induced by FAE. However, they also showed a difference in TMRM fluorescence as compared to untreated cells, indicating that mitochondrial membrane permeability is altered.

### FAE Triggerd Activation of Caspase 9

To examine the involvement of Caspases in FAE induced apoptosis, the activities of initiator Caspase 9 like (LEHDase) and effector Caspase 7/3 like (DEVDase) activities were investigated by fluorometric protease assay. MCF-7 cells were treated with FAE at IC_50_ value for 24, 36 and 48 h and Caspase activities were determined. FAE treatment resulted in a time dependent increase in the cleavage of Ac-LEHD-AFC (Acetyl-Leu-Glu-His-Asp-7-amino-4-trifluoromethyl coumarin) suggesting increased activity of Caspase 9 (Figure 5B). On 24 h treatment, no prominent cleavage of Ac-DEVD-AMC (substrate for Caspases 3/7; Acetyl-Asp-Glu-Val-Asp-7-Amino-4-methyl coumarin) was observed. Noticeable DEVDase activity occurred only at 36 h treatment with FAE (Figure 5C).

### FAE Induced Caspase Activation in Live Cell Model of Caspase Activation

**Figure 6:**
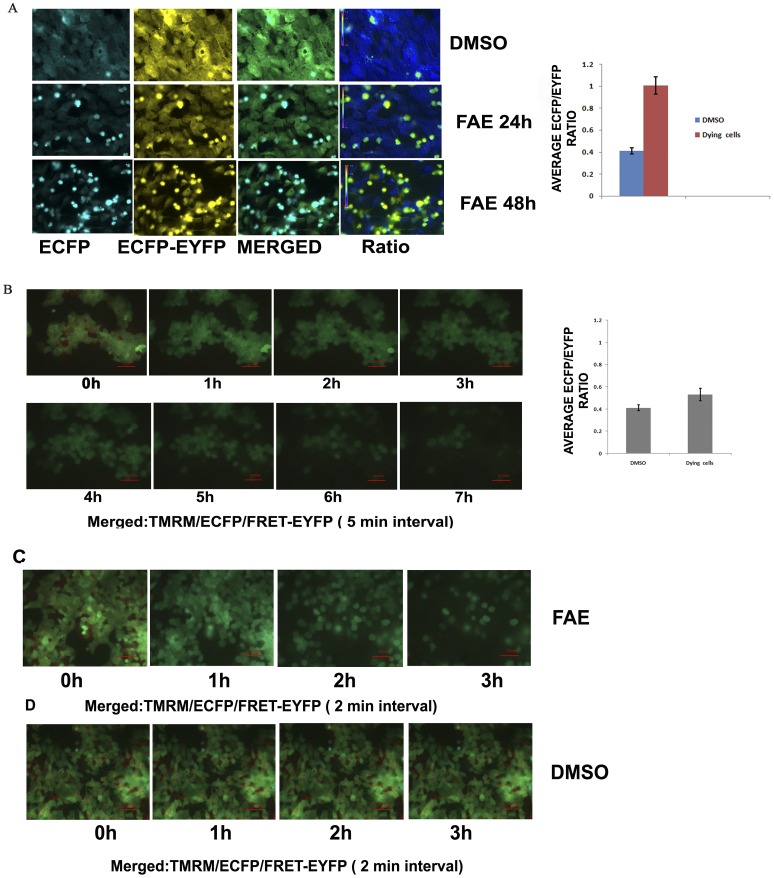
Caspase activation and photosensitizing effect of FAE. (**A**) MCF-7 cells expressing Caspase FRET probes were exposed to 100 µg/ml of FAE for 24 h and 48 h. The ECFP, EYFP-FRET channels and merged images for DMSO treated and FAE treated cells are shown. The ECFP/EYFP ratio image is also shown. The graph shown is the quantitative ratio change in DMSO and FAE treated dying cells as analysed in NIS elements software (n = 4). (**B**) The same cells were stained with TMRM and exposed to 100 µg/ml of FAE and imaged for TMRM, ECFP, EYFP-FRET in a time lapse mode at an interval of 5 mins as described. The merged image of TMRM, ECFP, EYFP-FRET from the time lapse images for the indicated time points are shown. The ECFP/EYFP ratio change in DMSO and FAE treated cells is shown as graph. The graph indicates the quantitative ratio change in DMSO and FAE-treated dying cells (Complete frame is shown as **Video S1**). (**C**) The FRET expressing cells were treated as above and the imaging was carried out as described with an interval of 2 mins. A representative image for the indicated time is shown (Complete frame is shown as V**ideo S2**). (**D**) The same cells treated with DMSO and imaged as described for C. A representative image for the indicated time is shown (Complete frame is shown as V**ideo S3**).

Above results indicated that FAE induced apoptosis primarily through mitochondria mediated intrinsic pathway involving Bax mediated mitochondrial permeabilization followed by Caspase 9 and Caspase 3/7 activation. Further, to substantiate the role of Caspases in FAE induced cell death, we have used a FRET (Fluorescence Resonance Energy Transfer) based live cell model as described earlier [28] to monitor activated Caspases in live cells. In brief, the breast cancer cell line expressing the FRET probe ECFP-DEVD-EYFP (Enhanced Cyan Fluorescent Protein- Caspase 3 cleavage sequence- Enhanced Yellow Fluorescent Protein) was exposed to FAE at 100 µg/ml and imaged in ratio mode. Upon Caspase activation, the FRET from the donor fluorophore to acceptor was lost with increase in ECFP and decrease in EYFP fluorescence. As shown in Figure 6A, the untreated control cells showed an increased acceptor fluorescence than the donor fluorescence that was reflected as decrease in ECFP/EYFP ratio (0.04) owing to increased energy transfer mediated acceptor fluorescence. However, in the treated wells, most cells showed an increased ratio to 1.005 indicating the loss of FRET owing to Caspase mediated cleavage of the linker DEVD placed in between donor and acceptor with increase in ECFP and decrease in EYFP fluorescence. The late apoptotic cells with noticeable cell shrinkage were seen as bright bodies in the image with sufficient ratio change in ratio image (Figure 6A).

### FAE Possessed Strong Photosensitizing Effect

For analysis of photosensitizing effect of FAE, the Caspase sensor expressing cells were seeded on 8- well-chambered glass bottom plates and stained with mitochondrial membrane potential specific fluorescent dye TMRM (Tetramethyl Rhodamine Methyl ester) as described earlier [28]. The cells were treated with 100 µg/ml of FAE and exposed to continuous imaging for TMRM as well as donor and acceptor fluorescence using CARV (BD Biosciences) confocal microscope for 24 h at a regular interval of 5 mins. As shown in the Figure 6B and Video S1, within 30 mins of imaging, the cells lost TMRM fluorescence indicating loss of mitochondrial membrane potential. Noticeable cell loss was evident as early as 60 mins that rapidly proceeded to complete loss of cells in the imaging plane by 7 h. Only a mild change in ECFP/EYFP ratio was noticed in these cells indicating partial Caspase activation (Figure 6B). Further, to substantiate the photosensitizing role of FAE, we have reduced the exposure interval to 2 mins with continuous imaging upto 200 frames. Interestingly, the cell loss correlated well with the frame number rather than the timing of the FAE treatment substantiating that the sensitizing effect was highly influenced by excitation light exposure rather than FAE treatment duration. The untreated cells exposed to same imaging conditions failed to show any change in ratio or cell loss for up to 24 h of imaging validating that the effect observed was caused by FAE (Figures 6B, C, D and Videos S2, S3). The cells also retained TMRM fluorescence indicating maintenance of mitochondrial membrane potential throughout the imaging period. Overall, the results confirmed that FAE possessed photosensitizing effect that was associated with rapid loss of mitochondrial transmembrane potential and partial Caspase activation.

### FAE Mediated Expression of Apoptotic Regulators

**Figure 7:**
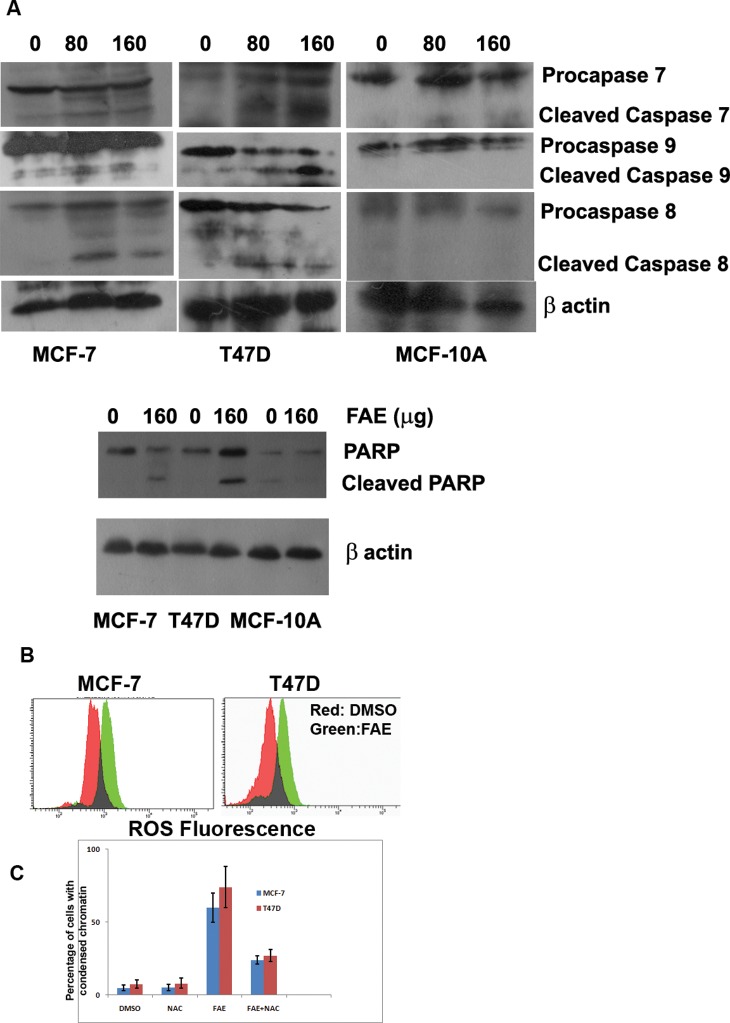
Effect of FAE on expression of apoptosis related proteins and ROS generation. (**A**) MCF-7 cells were treated with FAE (80, 160 µg/ml) for 48 h and harvested. Immunoblots were performed in whole cell extract using the indicated antibodies. The corresponding full-length and cleaved fragments are indicated. For PARP blot, cells were treated with 160 µg/ml of FAE for 48 h. (**B**) MCF-7 and T47D cells were treated with 100 µg/ml of FAE for 24 h. FAE induced ROS generation in MCF-7 as well as T47D cells than the DMSO control as indicated by increase in DCF-DA fluorescence in treated cells. (**C**) MCF-7 and T47D cells were treated with 100 µg/ml of FAE alone and after pre-treatment with NAC for a period of 24 h. As shown, pre-treatment of cells with the antioxidant NAC reduced the cell death induced by FAE. Percentage of cells with condensed chromatin for each group is shown as graph (N = 3).

Inorder to substantiate the importance of Caspase cascade behind the induction of apoptosis, we analysed the cleavage of important Caspases like Caspase 8, Caspase 7, Caspase 9 and also PARP (Poly (ADP-ribose) polymerase) in MCF-7, T47D and MCF10A. As shown in Figure 7A, FAE treatment at 80 and 160 µg/ml induced cleavage of Caspase 8, Caspase 7 and Caspase 9 in MCF-7 as well as in T47D. Cleavage of PARP was also observed in these cells. However, in MCF10A, only a mild cleavage was evident both for Caspases and PARP.

### FAE - induced Cell Death Involved Generation of ROS

The earlier results indicated that in most of the cell lines used, FAE could trigger loss of mitochondrial membrane integrity and condensation of chromatin possibly through Bax dependent manner. Moreover, upon live cell imaging we have accidently observed photosensitizing effect for the FAE extract in MCF-7 cell expressing caspase sensor probe with accelerated loss of mitochondrial membrane potential. Previously, it was reported that most photosensitizers induced cell death through the generation of ROS [29], one of the prominent triggers for Bax conformational activation. Therefore, we had analysed the ROS after treating the cells with 100 µg/ml of FAE for 24 h by FACS. As shown in Figure 7 B, FAE induced ROS generation in MCF-7 as well as T47D cells indicated by increase in 2′,7′-dichlorodihydrofluorescein diacetate (H_2_-DCF-DA) fluorescence in treated cells than the control. Pre-treatment of cells with the antioxidant N acetyl cysteine (NAC) reduced the cell death induced by FAE as shown in the chromatin condensation results (Figure 7 C). It again validated that generation of ROS within the cells was the primary trigger for apoptotic cell death and that might contribute for its photosensitizing activity.

## Discussion

Natural product extracts have been widely tested in the pharmaceutical industry and have been considered as a valuable source of new drugs [30]. As a means of identifying anti-cancer agents, the reverse pharmacology, or the ‘bedside’ to ‘bench’ approach has been explored that involves studying medicinal plants that have been traditionally used to treat various ailments. Various studies indicates that *Ficus* species are widely used in the management of various types of diseases like respiratory disorders, sexual disorders, central nervous system disorders (CNS), cardiovascular disorders (CVS), gastric problems, skin infections and diabetes etc [31], [6]. Most of the pharmacological studies were aimed on validating its traditional uses [32]. Although modern drug design emphasizes the development of single agents with specific targets, the fact that whole extract has been shown to be more efficacious than its individual components (a concept known as herbal synergy) suggests the limitations of this approach [3]. Killing of tumor cells through the induction of apoptosis is now recognised as a strategy for identifying anti-cancer drugs [33]. In the present report, we sought to determine the mechanisms by which the acetone fraction of *F.religiosa* leaf extract exerts its anti-proliferative effect in multiple breast cancer cells.

Anti-proliferative effect can be attributed to altered biochemical mechanisms including suppression of cell proliferation, growth arrest at the checkpoints of cell cycle and enhanced apoptosis induction [34]. By MTT, Sulforhodamine B assay and Trypan blue assay, we demonstrated the ability of FAE in suppressing proliferation and viability of multiple tumorigenic cell lines. In general, FAE showed less toxicity to non-tumorigenic mammary epithelial cells (Figure 1A). This observation was validated using Sulforhodamine B assay also (Figure 1B). FAE could induce morphological alterations, including membrane blebbing and shrinkage of MCF-7 cells, which were characteristic of apoptosis upon 48 and 72 h treatment (Figure 1E). FAE treatment inhibited colony formation even at a concentration of 40 µg/ml. At 40 µg/ml and 80 µg/ml, the number of colonies reduced by 51% and 42% of the original colony numbers respectively (Figure 1D). All these data suggested the potential of FAE in inhibiting the proliferation of breast cancer cells without inducing cell death in non-tumorigenic cells.

One of the main goals in the development of novel therapeutics for proliferative disorders is to generate agents that potentially inhibit cell cycle progression. Defects in cell cycle is a notable feature of many cancer cells allowing it to proliferate uncontrollably whereas in normal cells, cell cycle progression is regulated by cell cycle check points [35]. Majority of the chemopreventive agents currently used are capable of inducing either G_1_/S phase or G_2_/M phase arrest thereby preventing this uncontrolled division [36], [37]. The cell growth inhibition observed in the cell viability studies by FAE was associated with a moderate accumulation in the G_1_ phase of the cell cycle with corresponding decrease in S and G_2_ phase cells. Noticeable sub-G_0_ apoptotic population was evident in the histogram of MCF-7 cells treated with 100 µg/ml of FAE (Figures 2 A and B).

Apoptosis and associated cellular events have profound effects on the progression of benign to malignant neoplasm and are considered as important target for the therapy of various cancers [38]. It is a complex sequential process of genetically determined self-destruction that ultimately leads to the activation of proteases with certain substrate specificities, the Caspases and nucleases that produce membrane blebs, degrade DNA into nucleosome sized fragments and condensate cellular compartments [39]. Fluorescent microscopic imaging of FAE treated cells after Hoechst 33342 staining showed characteristic apoptotic morphology emitting bright fluorescence at 36 and 48 h FAE treatment (Figure 3A). This confirmed the ability of FAE to induce apoptosis. To further assess the extent of apoptosis induction by FAE, cells were analyzed by flow cytometry after staining with Annexin V and PI. The percentage of apoptosis quantified by flow cytometry analysis showed that 41.4% of the MCF-7 cells exhibited characteristics of cell apoptosis after 48 h treatment (Figures 3B and C).

The mitochondria-dependent pathway for apoptosis involves the release of Cytochrome c from mitochondria into the cytosol, either by the suppression of anti-apoptotic members or activation of pro-apoptotic members of the Bcl-2 family leading to the activation of Caspase 9 [40]. To visualize the movement of cytosolic Bax into mitochondria in live cells, we have employed a sensitive cell-based platform of MCF-7 cells expressing Bax protein fused with EGFP [26], [28]. Treatment of cells that express Bax-EGFP fusion protein with FAE resulted in Bax migration into mitochondria within 3 h, and after 27 h, most of the cells (93%) showed perinuclear granular fluorescence, indicating massive translocation of Bax to mitochondria (Figures 4A, B & C). Bax, being a strong pro-apoptotic protein, its over-expression sensitized the breast cancer cells to FAE induced cell death noticeably with early Bax translocation. This is consistent with earlier reports that Bax over-expression renders cells sensitive to drugs that act through Bax [25]. Moreover, FAE treatment resulted in considerable depletion of mitochondrial transmembrane potential in multiple breast cancer cells in a time dependent manner (Figure 5A). Even though the two normal cells used showed a diffused TMRM fluorescence, indicating partial membrane potential loss, the chromatin retained intact nuclear morphology, consistent with the MTT and Sulforhodamine B assay results. Collectively, the results suggested that FAE induced conformational changes in Bax and triggers mitochondria mediated apoptosis by release of mitochondrial membrane potential.

We further examined the possible involvement of Caspases in the induction of apoptosis by FAE on MCF-7 cells. The Caspase cascade system plays an important role in the induction, transduction and amplification of intracellular apoptotic signals. Generally, there are two pathways through which the Caspase family proteases can be activated: one is the death signal-induced, death receptor-mediated pathway; the other is the stress-induced, mitochondrion-mediated pathway (i.e. a Caspase 9-dependent pathway) [41]. MCF-7 breast carcinoma cells lack Caspase 3 owing to the functional deletion in the CASP-3 gene [42]. But studies have documented that Caspase 3 like proteases or Caspase 7 are involved in apoptosis induction by various chemopreventive agents in Caspase 3 deficient MCF-7 cells [43]. Our data showed that 36 and 48 h of treatment with FAE induced DEVDase (Caspase 3/7) activity which was less significant compared to LEHDase activity (Caspase 9) (p<0.05). FAE treatment triggered a marked elevation in LEHDase activity (Caspase 9) in a time dependent manner (p<0.001) (Figures 5B and C). Immunoblot analysis also revealed that FAE treatment induced a dose-dependent cleavage of Caspase 7, Caspase 8 and Caspase 9 in breast cancer cells, MCF-7 and T47D, suggesting the activation of mitochondria mediated pathway in apoptosis. However, FAE treatment failed to induce cleavage of Caspase 7, 8 and 9 in the non-tumorigenic breast epithelial cell, MCF10A. Both Caspase 3 and Caspase 7 have been reported to be responsible for PARP cleavage. These two Caspases recognize the same target sequence in substrate proteins for cleavage [44]. Later several reports suggest that PARP cleavage by Caspase 7 can occur in the absence of Caspase 3 activity [45]. Consistent with this, FAE treatment induced cleavage of PARP in MCF-7 and T47D cells (Figure 7A). Again, FAE failed to induce PARP cleavage in MCF10A cells. Further, we have employed a more sensitive system of Caspase activation, the FRET probe expressing cells to check the status of Caspase activation in MCF-7. Considerable number of cells showed Caspase activation after 24 h of treatment as shown by change in ECFP/EYFP ratio compared to untreated cells (Figure 6A).

Quite surprisingly we have noticed substantial photosensitizing effect for FAE as evidenced from the time lapse imaging of FRET probe expressing cells. Repeated excitation of the FRET probe, accelerated the cell death in a mitochondria dependent manner in treated cells. This cell death was accelerated with partial Caspase activation as evident from the mild change in ECFP/EYFP ratio (Figure 6B). Several studies have indicated that low power excitation in a continuous mode generate free radicals within the cells that most often will be neutralized by intracellular antioxidant machinery without affecting the viability of the cells [29]. But the untreated cells showed no evidence for cell death under the same imaging condition. The massive cell death observed in treated samples with continuous excitation may be due to the neutralization of antioxidant machinery of the cells by FAE promoting ROS mediated fast cell death. Consistent with this notion, we have observed increased intracellular ROS in treated cells and the pre-treatment of cells with the anti oxidant NAC reduced chromatin condensation induced by FAE (Figures 7B and C). Earlier studies suggest that intracellular ROS contribute for Bax activation during apoptotic cell death [27]. In addition, FAE could kill most of the breast cancer cell lines through the loss of mitochondrial membrane potential and condensation of chromatin. However, the two normal cells, endothelial cells and mammary epithelial cells were relatively less susceptible to mitochondrial membrane potential loss and chromatin condensation induced by FAE. All these data suggest the potential of FAE in inhibiting the proliferation and enhancing selective death of cancer cells without inducing cell death in non-tumorigenic cell line as well as normal primary cells. Additionally, we have shown that FAE possess strong photosensitizing activity involving accelerated mitochondrial transmembrane potential loss. Our studies using defined cellular models of apoptosis like Bax EGFP and caspase sensor cell lines also revealed that Bax mediated mitochondrial transmembrane potential loss contributed for the apoptotic cell death. Silencing of Bax in MCF-7 cells again supported the essential requirement of Bax in mediating FAE induced cell death (Figure 4D).

**Figure 8:**
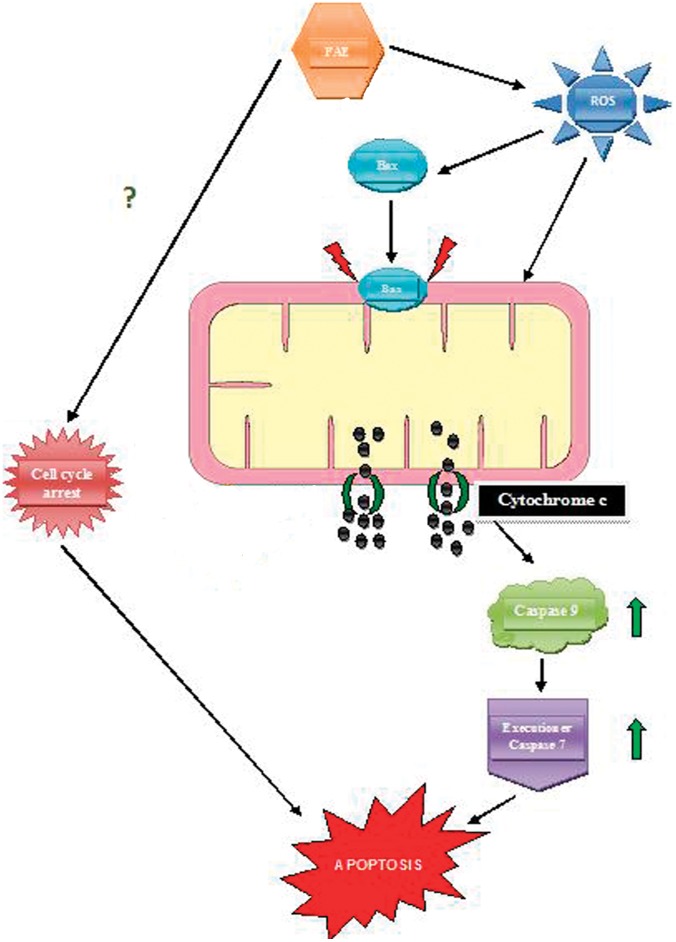
Proposed signalling pathway for FAE induced apoptosis. FAE induced conformational change in Bax through intracellular generation of ROS and triggered mitochondria mediated apoptosis involving loss of mitochondrial membrane potential. The release of mitochondrial Cytochrome c, which functions as an electron carrier in the respiratory chain, translocated to the cytosol, where it participated in the activation of Caspase 9 and 7, leading to apoptotic events in the cells. FAE also induced cell cycle arrest in G_1_ phase, and photosensitization, but the exact mechanism by which this is brought about has to be investigated.

The cancer cell specific apoptotic activity with moderate toxicity to normal cells and photosensitizing activity of FAE indicate that the plant extract could be a potential source for possible candidate drug identification. Photosensitizing agents are emerging as potential anti-cancer therapeutic agents to ensure the complete elimination of chemo resistant and radiation resistant cancers. Further studies are needed to purify the photosensitizing component(s) of FAE. To our knowledge, the results showed for the first time that FAE induced apoptosis in breast adenocarcinoma cell line and also it possessed strong photosensitizing effect. Based on the results obtained, we have proposed a mechanism for the FAE induced apoptosis (Figure 8) that involved ROS mediated Bax activation leading to mitochondrial potential loss followed by caspase activation. We conclude that, FAE inhibited the growth of MCF-7 human breast carcinoma cells and Bax induced mitochondria mediated apoptosis. FAE also possess strong photosensitizing effect on cancer cells that was mediated through rapid mitochondrial transmembrane potential collapse and partial Caspase activation. Further pre-clinical animal studies using purified compound(s) from this extract might help in identifying drug candidates that could replicate the biological activities of FAE.

## Materials and Methods

### Preparation of FAE and Phytochemical Analysis

Air-dried powdered leaves (10 g) were extracted using a Soxhlet apparatus successively with hexane, chloroform, acetone and methanol for 20 h in each solvent and then the extract was filtered and evaporated under reduced pressure. The final concentrate was recovered and dissolved in DMSO (dimethyl sulphoxide) (0.1% final volume) or stored in −80°C until use. Acetone extract showed minimum toxicity in non-tumorigenic cell line and maximum growth inhibitory activity in breast cancer cell line. So *F.religiosa* acetone leaf extract, referred as FAE was used for further experiments. The yield of the dried acetone extract obtained from the starting crude material was 8% (w/v). The freshly prepared crude extract (FAE) was qualitatively tested for the presence of alkaloids, flavonoids, phenols, saponins and tannins using standard procedures of analysis [21].

### Estimation of Total Flavonoids in FAE

Aluminium chloride colorimetric method was used for flavonoids estimation [22]. FAE (0.5 ml of 0.5 mg/ml) in 80% acetone was separately mixed with 0.1 ml of 10% aluminium chloride, 0.1 ml of 1 M potassium acetate and 4 ml of 80% acetone. After incubation at room temperature for 45 mins, the absorbance of the reaction mixture was measured at 415 nm with a double beam UV/Visible spectrophotometer (Perkin Elmer, USA). The reading was compared to a standard curve of prepared quercetin solutions and expressed as quercetin equivalents.

### Estimation of Total Phenols in FAE

The total phenolic contents of FAE were determined by Folin Ciocalteau method [23]. Briefly, FAE solution (0.5 mg/ml) was mixed with 1 ml Folin’s reagent and 0.8 ml Sodium Carbonate solution (1 M) and incubated for 30 mins. The absorbance at 765 nm was measured using a double beam UV/Visible spectrophotometer (Perkin Elmer, USA). The standard curve was prepared using 25–200 µg/ml solutions of gallic acid, a common reference compound for the estimation of phenols.

### Cell Culture

The breast cancer cell lines MCF-7, T47D, SKBr3 and MDAMB 231 and the normal breast epithelial cell, MCF-10A were obtained from the ATCC (American Type Culture collection, Manassas, VA). The mammary epithelial cells were procured form Lonza (Switzerland). Breast cancer cells were cultured in DMEM (Dulbecco’s Modified Eagle’s Medium) (Sigma, St. Louis, MO) supplemented with 10% FBS (Foetal Bovine Serum) (Gibco, NY, USA), penicillin (100 units/ml) and streptomycin (50 units/ml) in a humidified incubator with 5% CO_2_ atmosphere at 37°C**.** MCF-10A and mammary epithelial cells were cultured in MEBM (Mammary epithelial basal medium) supplemented with 20 ng/ml epidermal growth factor, 0.01 mg/ml insulin, 500 ng/ml hydrocortisone and 5% horse serum, penicillin 100 units/ml and streptomycin 50 units/ml with 5% CO_2_ atmosphere at 37°C DMSO (0.1% final volume) was used as vehicle control in all experiments. Human umbilical cord endothelial cells were isolated and maintained as described [46]. Cells were sub-cultured at 3 day intervals (80% confluent) using trypsin-EDTA solution in PBS buffer. Cells for the treatments were from passages 5–40.

### Cell Proliferation Assay

MTT assay was used to determine the effect of FAE on cell proliferation. This assay measures cell viability based on the ability of the mitochondrial reductase to convert the yellow tetrazolium MTT to a purple formazan dye. The intensity of purple colour developed is proportional to the number of live cells [47]. Cells were seeded at an initial density of 5×10^4^ cells/well in 96 well plates. After 24 h incubation, the medium was removed and replaced by 10% FBS medium containing 0, 20, 40, 80, 160 and 320 µg/ml of FAE. After incubation for 24, 48 and 72 h, the drug-containing medium was replaced by MTT solution (2 mg/ml) and incubated for 2 h at 37°C. The formazan crystals developed were solubilised in lysis buffer (20% sodium dodecyl sulphate in 50% dimethyl formamide) and incubated for 4 h at 37°C. The absorbance was measured spectrophotometrically at 570 nm with a microplate reader (Bio-Rad, San Diego, USA). The cell proliferation was calculated as CP =  (Optical density of drug exposed cells/Mean optical density of control cells) ×100.

### Trypan Blue Dye Exclusion Method

Dye exclusion stains like trypan blue can stain cells that lack an intact membrane. Viable cells with intact membrane are capable of excluding this dye [48]. MCF-7 cells were cultured in the presence of FAE (IC_50_ value) for 24, 48 and 72 h. After incubation, cells were washed with PBS (phosphate-buffered saline) and stained with 0.4% trypan blue for 3 mins. Unstained cells were counted using haemocytometer. The cell viability was calculated as cell viability (%)  =  total viable cells (number of unstained cells)/total number of cells (number stained and unstained cells) × 100.

### Sulforhodamine B Assay

Cytotoxicity was determined using a protein based viability test Sulforhodamine B assay. Briefly, drugs were added to the cells grown on 96 well plates with 100 µl of medium. After 72 h, plates were fixed in 10% trichloroacetic acid for 30 mins at 4°C, then washed in water and dried. 100 µl of 0.04% sulforhodamine B (Sigma, St. Louis, MO) in 1% acetic acid was added to each well and incubated for 15 mins. After removing excess stain by washing with 1% acetic acid four times and the plates were air dried. Finally the stain was solubilised using 10 mmol/l Tris base and read at 540 nm with a microplate reader (Bio-Rad, San Diego, USA). Each experiment was performed in quadruplicate and repeated at least three times. The relative cell viability (%) was calculated using the equation OD_T_/OD_C_×100% (where OD_T_ represents the absorbance of the treatment group and OD_C_ represents the absorbance of the control group) [49].

### Clonogenic Cell Survival Assay

Clonogenic cell survival assays were performed as previously described [50]. Briefly, MCF-7 cells were seeded in six well plates at 500 cells/well in phenol red free DMEM medium containing 10% FBS. After 12 h, cells were treated with 0, 40, 80, 160 and 320 µg/ml of FAE. The medium with FAE was changed after every 4 days. After 14 days of incubation, colonies were stained with 0.3% crystal violet solution for 2 mins, washed with PBS, air-dried and manually counted. Each experiment was performed in triplicates.

### Cell Cycle Analysis

Cells were seeded in six well plates at 2×10^5^ cells/well and cultured in 10% FBS medium with or without FAE. After treatment with FAE at 100 µg/ml for 24, 48 and 72 h, the cells were washed twice with ice cold PBS, collected by trypsinization and centrifuged. The pellets were re-suspended in 300 µl cold PBS and 700 µl cold 70% ethanol and incubated at 4°C. After centrifugation, the cells were washed and re-suspended in cold PBS, containing RNase (5 mg/ml) and incubated at 37°C for 30 mins. Finally, propidium iodide (1 mg/ml) was added and incubated in the dark for 10 mins and cells were analyzed using FACS Aria 1 (Becton Dickinson, San Jose, CA). Cell distribution in the different phases of the cell cycle was analyzed with BD Diva software.

### Analysis of Apoptotic Cells

The AnnexinV-FITC apoptosis detection kit (Sigma-Aldrich Inc., USA) was used for the detection of apoptotic cells as per manufacturer’s protocol. Briefly, cells were incubated in the presence of FAE at IC_50_ value for 24, 36 and 48 h. After treatment, cells were washed with cold PBS followed by trypsinization. From the cell suspension, 1×10^6^ cells were re-suspended in 1× binding buffer and then incubated with 5 µl of Annexin V-FITC and 10 µl of PI solution and incubated for 10 mins in dark. Fluorescence of the cells was determined immediately using flow cytometry (Becton Dickinson, San Jose, CA). Cells in the early apoptosis will stain with AnnexinV-FITC conjugate alone [51]. Apoptotic cell population was further detected using Hoechst 33342 staining. After treatment with FAE at IC_50_ value for 24, 48 and 72 h, cells were fixed with 4% paraformaldehyde for 10 mins at room temperature, stained with Hoechst 33342 (5 µg/ml) for 20 mins at 37°C in the dark, and visualised under fluorescence microscope (Eclipse E-600, Nikon, NY, USA) utilizing a 350 nm excitation and a 460 nm emission filter. The large nuclei of normal cells stained faintly with Hoechst 33342, whereas condensed chromatin of apoptotic nuclei stained brightly.

### Transfection Studies and Live Cell Analysis of Bax Translocation to Mitochondria

The expression vector for Bax-EGFP was provided by Dr. Clark Distelhorst. The breast cancer cell line MCF-7 was transfected with Bax-EGFP plasmids using lipofectamine as per the manufacturer’s instruction. After 12 h of transfection, the cells were maintained in G418 selection medium for 2–4 weeks. The EGFP expressing clones were expanded and transfected with Mito DsRed vector (Clonetech, USA) to visualize mitochondria.

### Bax Translocation Analysis by Microscopy

The MCF-7 cells expressing Bax-EGFP and Mito DsRed were seeded in 96 well glass bottom plate (BD, USA) with low density and after 48 h, treated with 100 µg/ml of FAE. For quantitative Bax translocation analysis, images were taken using BD Pathway Bioimager 435 (Becton Dickinson, USA) at 3, 18 and 27 h by setting Montage (2×2) and specific Macro using AttoVision™ software. The filter combination used for imaging EGFP consists of Ex 472±15 and Em 520±17 nm filters. The DsRed was excited with 540**±**20 nm and emission was collected using 592±22 nm filter. The representative images collected at indicated time points were used for analysing the percentage of positive cells with Bax-EGFP at mitochondria compared to total in the field. For visualization of Bax aggregates on mitochondria in high magnification, cells were imaged with 40× 0.95 NA objective using Tie Microscope (Nikon). The images were acquired using Retiga Exi camera and NIS element software (Nikon).

### Silencing of Bax by siRNA

MCF-7 cells were seeded on 6 well plates at a density of 2×10^5^ cells and incubated for 48 h in complete medium. The cells were transfected with 8 µl of siRNA specific for human Bax (sc-29212, Santacruz) or control siRNA (sc-37007, Santacruz) using the siRNA transfection reagent (sc-29528, Santacruz) as per the manufacturer’s instruction. 12 h after transfection, transfection medium was replaced with fresh medium containing serum and allowed to grow for 24 h. Whole cell extract was prepared 48 h after transfection for analysis of silencing efficiency by western blot using antibody against Bax (Santacruz, sc-493). In a separate experiment, cells after 24 h of transfection were either treated with DMSO or FAE 100 µg/ml for 48 h. Then the cells were processed for chromatin condensation analysis after staining the cells with Hoechst 33342.

### Live Cell Caspase Analysis by FRET

The breast cancer cell line MCF-7 expressing FRET based Caspase sensor, ECFP-DEVD-EYFP was employed for monitoring Caspase activation in live cells. The development of the stable cell line and imaging conditions were reported previously [26]. For the analysis of Caspase activation, the above cell line was seeded at a density of 2000 cells/well in 96 well plates and allowed to grow for 24 h. The cells were incubated with increasing concentration of the extract for 24 h followed by ratio imaging using CARV confocal imager (BD Biosciences) as described previously [26], [28]. For ratio change analysis and quantification, images were exported to NIS element software (Nikon) and the regions of interest were drawn to quantify the ratio in cells.

### Analysis of Photosensitizing Effect of FAE

In order to study the photosensitizing effect of FAE, MCF-7 cells expressing Caspase sensor FRET probe were grown on 8 well chambered cover glass for 24 h. Then the cells were stained with 200 nM of TMRM for 10 mins. The cells were treated with FAE containing 20 nM TMRM and exposed to continuous imaging for TMRM, ECFP and FRET EYFP at an interval of 5 mins for 24 h. The excitation light intensity was maintained at 20% from the 120 W metal halide lamp with the help of intensity iris control unit of CARV11 confocal microscope. The cells were treated with DMSO only and imaged at the same imaging parameters served as control. For further substantiating the photosensitizing effect of FAE, the imaging interval was reduced to 2 mins with a total frame of 200.

### Detection of Caspase Catalytic Activities

The activities of Caspase 3/7 (DEVDase) and Caspase 9 (LEHDase) were studied using the Caspase fluorogenic substrates (Calbiochem). Assays were based on fluorometric measurement of fluorescent 7-amino-4-trifluoromethyl coumarin (AFC) after cleavage from the AFC-labeled peptide substrates Ac-DEVD-AMC for Caspase 3/7 and Ac-LEHD-AFC for Caspase 9. Briefly, after being treated with FAE at IC_50_ value for 24, 36 and 48 h, cell lysate was prepared and protein concentration was determined using Bradford’s assay. 50 µg of each cell lysate was re-suspended in 50 µl of cell lysis buffer and incubated with 5 µl of 1 mM stock of fluorescently labelled Caspase substrate at 37°C for 1–2 h. The release of cleaved substrate was measured with a fluorometric plate reader (Tecan Infinite M 200, Austria) at an excitation wavelength of 400 nm and an emission wavelength of 505 nm. Experiments were performed in triplicates.

### Evaluation of Mitochondrial Membrane Potential and Chromatin Condensation

In order to detect mitochondrial membrane potential loss and chromatin condensation simultaneously, we have employed dual staining of cells with mitochondrial membrane potential specific dye TMRM and nuclear stain Hoechst 33342. Briefly, the cells after treatment with FAE were stained with 50 nM of TMRM and 0.5 µg/ml of Hoechst 33342 for 15 mins. Then the cells were imaged under fluorescent microscope using DAPI and Rhodamine filter sets using 40× objective. The images were captured with Retiga Exi camera using NIS (Photometrics) element software (Nikon).

### Western Blot

Cells were washed with PBS and lysed using RIPA (Radioimmunoprecipitation assay) buffer (150 mM NaCl, 1% NP-40, 0.5% Sodium deoxycholate, 0.1% SDS, 50 mM Tris-HCl pH 7.4) containing protease inhibitor cocktail (Sigma-Aldrich Inc., USA). Briefly, equal amount of protein as determined by Bradford assay was subjected to SDS-PAGE, followed by transfer to nitrocellulose membrane (Millipore, MA). The membrane was then blocked in 5% powdered non-fat milk Tris solution for 1 h. Membrane was then incubated overnight with primary antibody, followed by incubation with species-specific horseradish peroxide (HRP) conjugated secondary antibody (1∶5000, Santa Cruz) at room temperature for 1 h. Protein bands were visualised on X-ray film using ECL-plus reagents (Amersham, NJ). The primary antibodies were: Caspase 8 (1∶500, Santa Cruz), Caspase 9 (1∶1000, Cell signalling), PARP (1∶1000, Cell signalling), Caspase 7 (1∶500, Cell signalling), Bax (1∶200, Santa Cruz) and β-actin (1∶5000, Sigma Aldrich Inc., USA).

### Detection of Intracellular ROS

For detection of intracellular ROS generation, ROS specific fluorescent probe H_2_-DCF-DA (Invitrogen) was employed as per the manufacturer’s protocol. Briefly, after treating the cells with indicated concentration of drug, the cells were removed by mild trypsinisation and immediately washed with serum containing medium. Then the cells were loaded with 10 µM H_2_-DCF-DA, incubated at 37°C for 30 min, in serum free OptiMEM and immediately analyzed by flow cytometry (FACS Aria 1 Becton Dickinson, San Jose, CA). For ROS inhibition analysis, cells were pre-treated with 5 mM NAC for 1 h followed by drug treatment. The cells were stained with Hoechst 0.5 µg/ml for 10 mins and imaged under UV filter to visualize the condensation of chromatin. Cells with condensed chromatin were counted from 200 cells per well to calculate the percentage of cells with condensed chromatin (n = 3).

### Statistical Analysis

Data were expressed as mean ± SD. One-way analysis of variance (ANOVA) was used to assess the significant differences between the treatment groups. Statistical analysis was performed using SPSS statistical software package (version 16.0). A probability of p≤0.05 (*) was considered significant.

## Supporting Information

Video S1 **MCF-7 cells expressing Caspase FRET probe was stained with TMRM and exposed to 100 µg/ml of FAE and imaged for TMRM, ECFP, EYFP-FRET in a time-lapse mode at an interval of 5 mins as described.** The merged image of TMRM, ECFP, EYFP-FRET from the time lapse images are shown.
(MP4)

Video S2 **MCF-7 cells expressing Caspase FRET probe was stained with TMRM and exposed to 100 µg/ml of FAE and imaged for TMRM, ECFP, EYFP-FRET in a time-lapse mode at an interval of 2 mins as described.** The merged image of TMRM, ECFP, EYFP-FRET from the time lapse images are shown.
(MP4)

Video S3 **MCF-7 cells expressing Caspase FRET probe was stained with TMRM and exposed to DMSO only and imaged for TMRM, ECFP, EYFP-FRET in a time-lapse mode at an interval of 2 mins as described.** The merged image of TMRM, ECFP, EYFP-FRET from the time lapse images are shown.
(MP4)
