# Correction: Bax Translocation Mediated Mitochondrial Apoptosis and Caspase Dependent Photosensitizing Effect of *Ficus religiosa* on Cancer Cells

**DOI:** 10.1371/annotation/ab341223-61ed-4b19-95c7-21455c321e06

**Published:** 2012-10-31

**Authors:** Jazir Haneef, Parvathy M, Santhosh Kumar Thankayyan R, Hima Sithul, Sreeja Sreeharshan

The second author's name was spelled incorrectly. The correct name is: Muraleedharan Parvathy.

The correct citation is: Haneef J, Parvathy M, Thankayyan R SK, Sithul H, Sreeharshan S (2012) Bax Translocation Mediated Mitochondrial Apoptosis and Caspase Dependent Photosensitizing Effect of Ficus religiosa on Cancer Cells. PLoS ONE 7(7): e40055. doi:10.1371/journal.pone.0040055 

